# On the Calculations of Electron Impact Ionization Cross-Sections for Selected Nucleosides and Deoxyribose Molecules

**DOI:** 10.3390/molecules31111964

**Published:** 2026-06-05

**Authors:** Paweł Możejko

**Affiliations:** Institute of Physics and Applied Computer Science, Faculty of Applied Physics and Mathematics, Gdańsk University of Technology, ul. Gabriela Narutowicza 11/12, 80-233 Gdańsk, Poland; paw@pg.edu.pl; Tel.: +48-583-472-921

**Keywords:** electron impact ionization, binary-encounter-Bethe model, Uridine, Cytidine, Thymidine, Adenosine, Guanosine, deoxyribose, electron scattering

## Abstract

Total cross-sections for the single electron impact ionization of deoxyribose (C_5_H_10_O_4_), Uridine (C_9_H_12_N_2_O_6_), Thymidine (C_10_H_14_N_2_O_5_), Cytidine (C_9_H_13_N_3_O_5_), Adenosine (C_10_H_13_N_4_O_4_), and Guanosine (C_10_H_13_N_5_O_5_) have been calculated using the binary-encounter-Bethe model from the first ionization threshold up to 4 keV. Electronic structure calculations of the studied targets have been performed at the Hartree–Fock (H-F) level using quantum chemical methods, including the outer valence Green function (OVGF) method, in order to obtain all the necessary physical input parameters for the BEB method. The possibility and feasibility of estimating the ionization cross-sections of larger DNA building blocks, such as nucleosides, based on the sum of the ionization cross-sections of DNA bases and simple sugar analogs, such as α-tetrahydrofurfuryl alcohol or deoxyribose, are also discussed.

## 1. Introduction

Since the discovery that low-energy electrons (0–20 eV) can cause damage to DNA and RNA, including single- and double-strand breaks [1], a finding that was later confirmed by subsequent experiments [2,3,4], intensive experimental [5,6,7] and theoretical [6,8] research is being conducted on the scattering of low- and medium-energy electrons from the simple and more complex building blocks of DNA and RNA, as well as their analogs. Such secondary electrons are produced by the interaction of primary high-energy ionizing radiation with matter. On average, for every 1 MeV of energy deposited in the medium, $10^{4}$ secondary electrons are produced with energies ranging from 0 to 100 eV [9]. These secondary electrons are not only produced by the ionization process itself but can also lead to secondary ionization of the medium. Due to their energy, this secondary ionization can be highly efficient, given that for most molecules, the maximum ionization efficiency in collisions with electrons occurs at energies in the range of 70–90 eV [10]. Therefore, in simulations, including Monte Carlo simulations, of the interaction of primary ionizing radiation (α, $\beta^{\pm }$, γ, etc.) with biological tissue, and, in future radiation therapy planning, processes involving the interaction of secondary electrons (including electron impact ionization) with medium targets must be taken into account. Indeed, some Monte Carlo simulation programs for the interaction of high-energy radiation with matter already often include modules dedicated to secondary particle interactions. The Geant4-DNA toolkit [11,12,13], for example, is already equipped with modules for describing the interactions of secondary electrons with the biological environment in many scattering channels including elastic electron scattering (7.4 eV–1 MeV), electron impact electronic excitation (9 eV–1 MeV), electron impact vibrational excitation (2 eV–100 eV), electron impact ionization (11 eV–1 MeV), and electron attachment (4 eV–13 eV). The data required for such simulations must, on the one hand, be highly reliable and, on the other hand, be as easy as possible to generate during the simulation. Therefore, there is a constant need to develop simple models that describe the interaction of low- and intermediate-energy electrons with biomaterials and provide a dataset obtained using various often more advanced methods to verify and validate these models.

**Figure 1:**
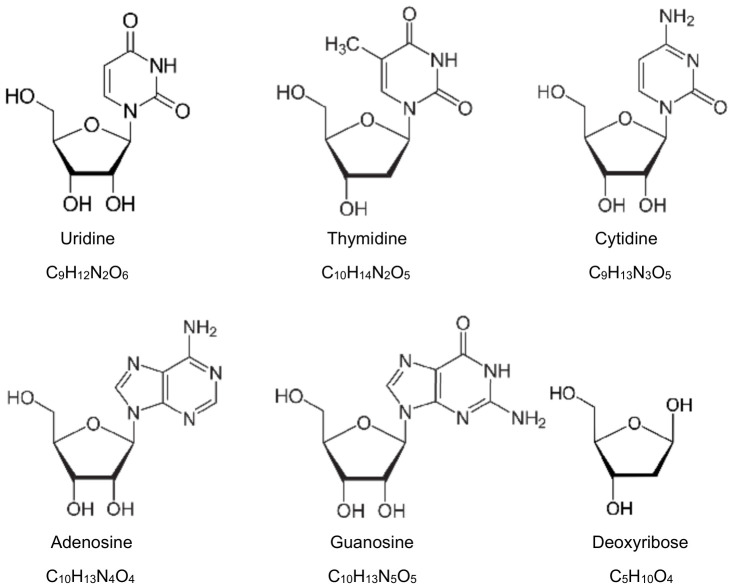
Schematic drawing of studied targets.

The primary objective of this work is to calculate the total cross-sections for single electron impact-induced ionization of deoxyribose (C_5_H_10_O_4_) and selected nucleosides, i.e., Uridine (C_9_H_12_N_2_O_6_), Thymidine (C_10_H_14_N_2_O_5_), Cytidine (C_9_H_13_N_3_O_5_), Adenosine (C_10_H_13_N_4_O_4_), and Guanosine (C_10_H_13_N_5_O_5_), over a wide range of incident electron energies, from the first ionization threshold up to 4 keV. Another objective of this work is to analyze the possibility of estimating the ionization cross-sections of more complex DNA and RNA fragments based on the cross-sections determined for their basic building blocks, e.g., DNA bases and simple sugar analogs such as deoxyribose or α-tetrahydrofurfuryl alcohol (C_5_H_10_O_2_). The structures of the studied compounds are shown schematically in Figure 1. Deoxyribose is a monosaccharide that can be considered as a deoxy sugar derived from the sugar ribose by loss of a hydroxy group. Nucleosides can generally be thought of as nucleotides without a phosphate group. A nucleoside consists simply of a nucleobase and a five-carbon sugar (ribose or 2′-deoxyribose).

Given the importance of low-energy electron interactions with DNA and RNA components, research on electron scattering on nucleosides has been relatively scarce to date. Studies of the fragmentation of Uridine and Thymidine upon electron impact ionization have been performed using a crossed electron-molecule beam apparatus combined with a quadrupole mass spectrometer [14]. The Schwinger multichannel method with the static-exchange approximation has been employed in integral elastic cross-section calculations for low-energy (0–20 eV) electron collisions with selected nucleosides [15]. Absolute cross-sections for vibrational and electronic excitations by low-energy electron scattering from condensed Thymidine on a multilayer film of Ar held at 20 K have been measured using high-resolution electron energy loss spectroscopy [16,17]. A preliminary version of our electron impact ionization cross-section calculations for nucleosides preformed using the binary-encounter-Bethe (BEB) method for electron energies ranging from the ionization threshold up to 200 eV was presented at the 31^*st*^ *International Conference on Photonic, Electronic and Atomic Collisions* [18]. More recently, a set of electron impact cross-sections for selected scattering channels, including inelastic, ionization, electronic excitation, elastic, and total cross-sections, has been computed using a complex optical potential approach [19]. Most recently, the interactions of 25 eV electrons with four types of nucleosides (Cytidine, Thymidine, Adenosine, and Guanosine) were studied using X-ray photoelectron spectroscopy (XPS) [20]. To the best of our knowledge, there are no published data on the experimental electron impact ionization cross-sections of nucleosides. Please note that the relatively small number of available experimental cross-sections for electron scattering on nucleosides is due to the fact that typical experiments to determine these cross-sections are performed in the gas phase. In contrast, nucleosides have relatively high melting points (mp) under normal conditions. It is therefore difficult to obtain a stable gaseous sample of such compounds. Consequently, it becomes much more difficult to maintain a constant concentration of target molecules in the collision region, which can lead to a significant increase in experimental uncertainty. For example, the melting points of Thymidine and Cytidine are 185 °C and 210 °C, respectively.

There have also been few experimental and theoretical studies on the interaction of low-energy electrons with deoxyribose so far. The cross-sections for differential and elastic scattering of low-energy electrons (0–50 eV) by deoxyribose have been studied using the Schwinger multichannel method with the static-exchange approximation [21]. The total electron impact ionization cross-section for deoxyribose molecule has been calculated using improved binary-encounter-dipole (BED) formalism for energies ranging from the ionization threshold to $10^{4}$ eV [22]. Doubly differential and integral cross-sections for electron elastic scattering on deoxyribose have been numerically calculated using the independent atom method [23].

The calculated cross-sections for the electron ionization of deoxyribose and selected nucleosides, as well as details of the method used, are presented and discussed in the following two sections.

## 2. Results and Discussion

**Figure 2:**
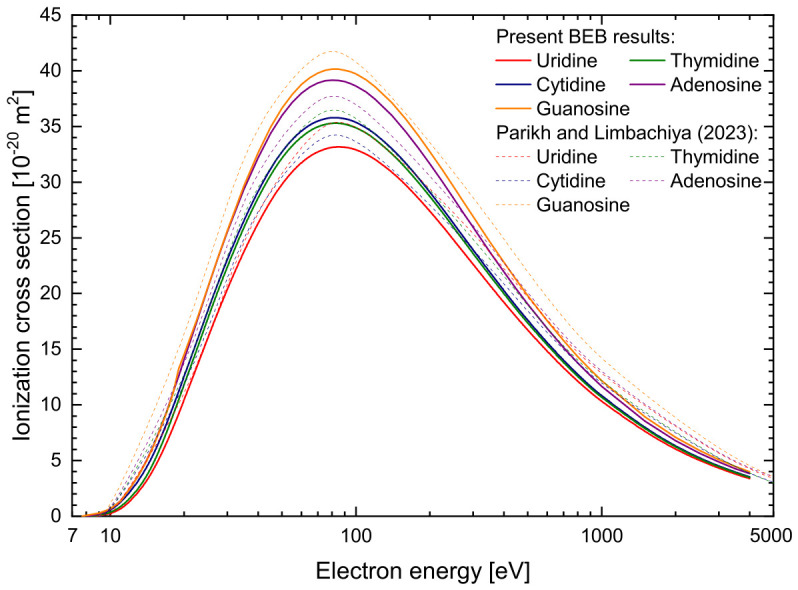
Comparison of the ionization cross-sections due to electron impact on studied nucleosides. Present BEB results: red solid line, Uridine; green solid line, Thymidine; blue solid line, Cytidine; purple solid line, Adenosine; orange solid line, Guanosine. Ionization cross-sections obtained with complex optical potential approach [19]: red dash line, Uridine; green dash line, Thymidine; blue dash line, Cytidine; purple dash line, Adenosine; orange dash line, Guanosine.

The calculated cross-sections for electron impact ionization of deoxyribose, C_5_H_10_O_4_, and selected nucleosides are presented in numerical form, for energies ranging from the ionization threshold to 4 keV in Table 1. The ionization cross-sections computed in this work, for Uridine, C_9_H_12_N_2_O_6_, Thymidine, C_10_H_14_N_2_O_5_, Cytidine, C_9_H_13_N_3_O_5_, Adenosine, C_10_H_13_N_4_O_4_, and Guanosine, C_10_H_13_N_5_O_5_, are shown in Figure 2. They are compared with the results of ionization cross-section calculations obtained with the complex optical potential method [19]. To date, no data are available on the experimental cross-section for the electron impact ionization of nucleosides.

The first ionization thresholds calculated in this study for Uridine, Thymidine, and Cytidine are 8.627 eV, 8.352 eV, and 8.368 eV, respectively. The ionization thresholds for Adenosine and Guanosine are located slightly lower, at 8.057 eV and 7.671 eV, respectively. These ionization thresholds are slightly lower (up to 0.4 eV) than the vertical ionization potentials obtained by ultraviolet photoelectron spectroscopy [24]. The latter are 9.0 eV for Uridine, 8.7 eV for Thymidine, 8.6 eV for Cytidine, 8.4 eV for Adenosine, and 8.0 eV for Guanosine [24]. The present ionization thresholds for Uridine and Thymidine are also in satisfactory agreement with the appearance energies measured using a crossed electron-molecule beam apparatus combined with a quadrupole mass spectrometer [14]. The measured appearance energy values are 9.05±0.15 and 8.80±0.17 [14] for Uridine and Thymidine parent cations, respectively.

Among the nucleosides studied here, Guanosine has the largest ionization cross-section, while Uridine has the smallest. In fact, across the entire range of energies studied and excluding behavior near the ionization threshold, the ionization cross-sections satisfy the inequality $\sigma_{\mathrm{Uridine}}^{\mathrm{ion}}<\sigma_{\mathrm{Thymidine}}^{\mathrm{ion}}<\sigma_{\mathrm{Cytidine}}^{\mathrm{ion}}<\sigma_{\mathrm{Adenosine}}^{\mathrm{ion}}<\sigma_{\mathrm{Guanosine}}^{\mathrm{ion}}$. This generally reflects the dependence of the ionization cross-section on the size of the molecule or, more precisely, the molecular polarizability volume, at least within the range of electron energies for which the cross-section reaches its maximum value [25,26]. Furthermore, the largest contribution to the ionization process comes from the ionization of valence electrons. The lower the ionization energy for a given orbital, the greater its contribution to the total ionization cross-section. Consequently, the observed relationship between the ionization cross-sections of the studied nucleosides is a function of their electronic structure, including the relationship between their ionization thresholds. The maxima in the ICS are located in the 80–85 eV range. Specifically, for Uridine, the maximum of $33.18\times 10^{-20}$ m^2^ is located at 85 eV, while for Thymidine, the maximum of $35.29\times 10^{-20}$ m^2^ is visible at 80 eV. For Cytidine, the maximum of $35.80\times 10^{-20}$ m^2^ is located at 80 eV. For Adenosine, the maximum is equal to $39.17\times 10^{-20}$ m^2^ at 80 eV, while for Guanosine, it is equal to $40.14\times 10^{-20}$ m^2^ at 85 eV. These maximum values are in fairly good agreement with predictions based on the polarizability correlation model, which relates the maximum of the ionization cross-section to the molecular static electronic polarizability volume [25,26]. The values predicted by this model for nucleosides are $31.70\times 10^{-20}$ m^2^ for Uridine, $34.37\times 10^{-20}$ m^2^ for Thymidine, $33.26\times 10^{-20}$ m^2^ for Cytidine, $37.44\times 10^{-20}$ m^2^ for Adenosine, and $38.96\times 10^{-20}$ m^2^ for Guanosine [27].

The results of our calculations are in good agreement with the data obtained using the complex optical potential method [19]. For Uridine, Thymidine, and Guanosine, the results of the present calculations are lower than those obtained by the complex optical potential method. For Cytidine and Adenosine, however, the relationship is reversed. The behavior near the ionization threshold and the dynamics of the increase in the ionization cross-sections up to the region of the maximum are nearly identical for both datasets. It should be noted that discrepancies in the vicinity of the ionization maximum do not exceed 15%. At higher collision energies, i.e., above 300 eV, the results obtained using the complex optical potential method [19] are systematically higher than the present results. These differences are slightly larger but still do not exceed 20%. It is worth noting that these discrepancies are within the accuracy of the BEB method (±15%) [28,29,30] that we used.

As there are no experimental data on the ionization cross-sections of nucleosides, the predicted reliability of the calculated cross-sections will be assessed in relation to data for the tetrahydrofuran (THF) molecule. THF can be treated as a benchmark molecule due to the large amount of available experimental and computational data [31]. Observed discrepancies between the total ionization cross-section measured by combining simultaneous electron and ion measurements with an analysis of the time-of-flight of ion-induced fragmentation [32] and the other results [27,33,34,35] are up to 10%. These discrepancies may be related to the experimental methods employed, differences in the detection efficiency of the ions, and the stability of the target density number. It is also noteworthy that there is the excellent agreement (to within 6%) between the experimental total cross-section for electron impact ionization of tetrahydrofuran, obtained by combining simultaneous electron and ion measurements with an analysis of the time-of-flight of ion-induced fragmentation, and the results of calculations obtained using the binary-encounter-Bethe method [36]. This finding is consistent with the well-documented strong correlation between the total ionization cross-sections calculated using the BEB method and the measurement results [30].

**Figure 3:**
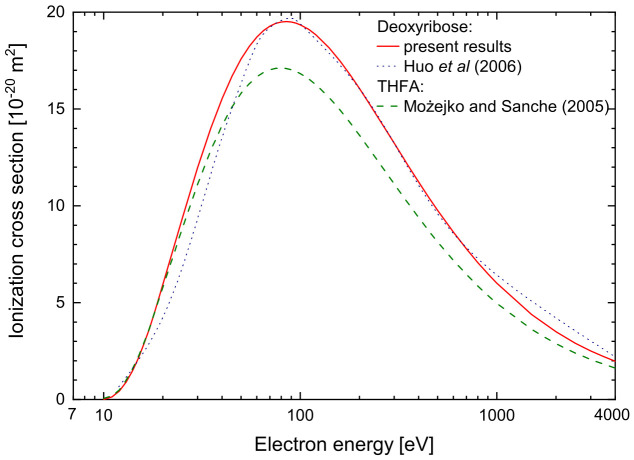
The electron impact ionization cross-section for deoxyribose, C_5_H_10_O_4_, molecule: solid red line, present BEB calculations; dotted blue line, BED calculations [22]. For comparison, ICSs for α-tetrahydrofurfuryl alcohol (THFA), C_5_H_10_O_2_, are also shown: dashed green line, BEB calculation [36].

Figure 3 shows the computed electron impact ionization cross-section for deoxyribose, along with previous data obtained using the complex optical potential approach [19]. Additionally, Figure 3 shows the cross-section for electron-induced ionization of α-tetrahydrofurfuryl alcohol, C_5_H_10_O_2_, molecule [36], which, along with tetrahydrofuran, C_4_H_8_O, is often considered an analog of DNA sugar. The calculated value of the first ionization threshold for deoxyribose is 9.992 eV. This value is slightly lower than the previously predicted value of 10.6 eV [22]. The maximum value of the present electron impact ionization cross-section for deoxyribose is $19.52\times 10^{-20}$ m^2^ at 80 eV.

The present results are in good agreement with the shape of the data obtained using the improved BED method [22]. However, differences can be seen in the cross-section behavior near the ionization threshold. At these low energies, the cross-sections obtained using the improved BED method increase much more slowly than our data, with the increasing energy of the ionizing electron. At 30 eV, the present results are approximately 30% higher than those calculated using the improved BED method [22]. Both datasets are in very good agreement in the region of the ionization maximum. Starting at 60 eV, the differences between the two datasets are essentially negligible. It is worth noting that the total ionization cross-sections for α-tetrahydrofurfuryl alcohol [36], a molecule often treated as an analog of DNA sugar, calculated also using the BEB method, are lower than those obtained for deoxyribose across the entire range of energies compared. These differences amount to approximately 15% near the ionization maximum and increase to around 30% for higher collision energies.

**Figure 4:**
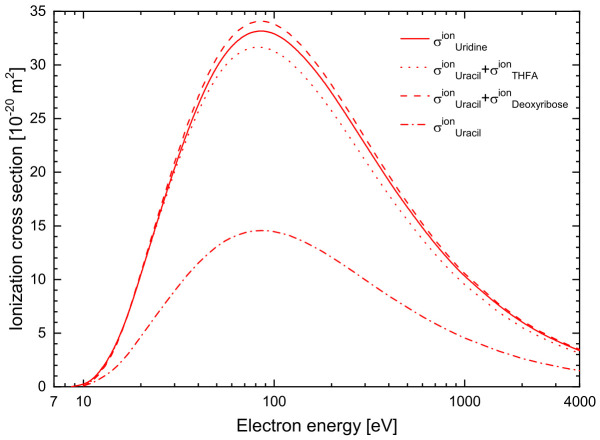
Comparison of the electron impact ionization cross-section for Uridine and uracil molecules. Estimated ionization cross-sections for Uridine, obtained as the sum of the ionization cross-sections for uracil and α-tetrahydrofurfuryl alcohol, and for uracil and deoxyribose, are also presented.

Figure 4 shows a comparison of the calculated total ionization cross-section for Uridine with the cross-section for uracil [37] ionization. The figure also compares the total ionization cross-sections obtained as the sum of the cross-sections for uracil [37] and α-tetrahydrofurfuryl alcohol [36] ionization, and for uracil [37] and deoxyribose ionization. This method of estimating cross-sections for larger molecules by summing the cross-sections of their molecular analogs can be used for compounds for which direct calculations may be difficult for various reasons. For instance, the ionization cross-section for the sugar phosphate DNA backbone unit has been approximated as the sum of electron impact ionization cross-sections of phosphoric acid and THFA [36] and alternatively as the sum of the ionization cross-sections for phosphate and deoxyribose [22].

**Figure 5:**
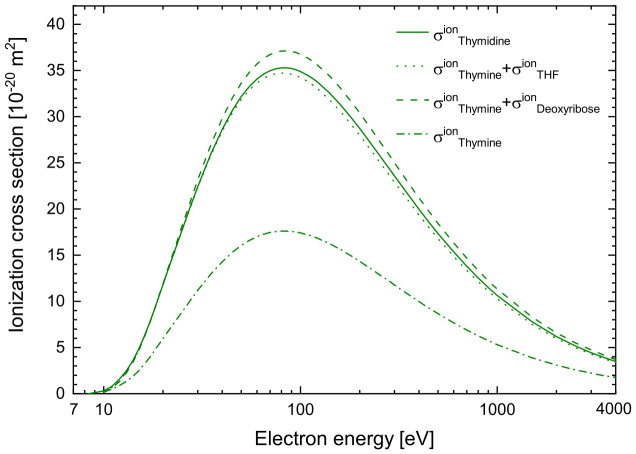
Comparison of the electron impact ionization cross-section for Thymidine and thymine molecules. Estimated ionization cross-sections for Thymidine, obtained as the sum of the ionization cross-sections for thymine and α-tetrahydrofurfuryl alcohol, and for thymine and deoxyribose, are also presented.

**Figure 6:**
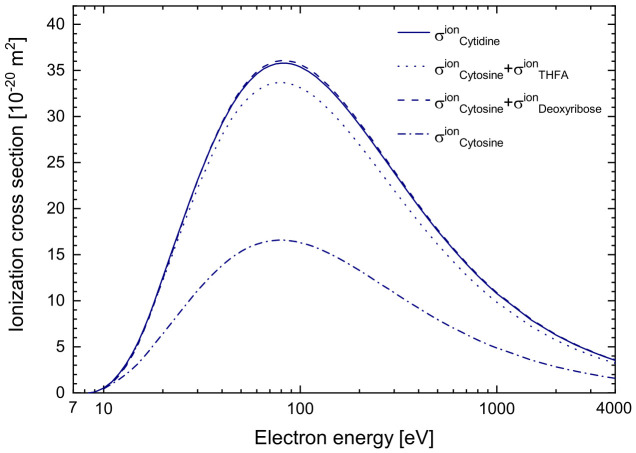
Comparison of the electron impact ionization cross-section for Cytidine and cytosine molecules. Estimated ionization cross-sections for Cytidine, obtained as the sum of the ionization cross-sections for cytosine and α-tetrahydrofurfuryl alcohol, and for cytosine and deoxyribose, are also presented.

**Figure 7:**
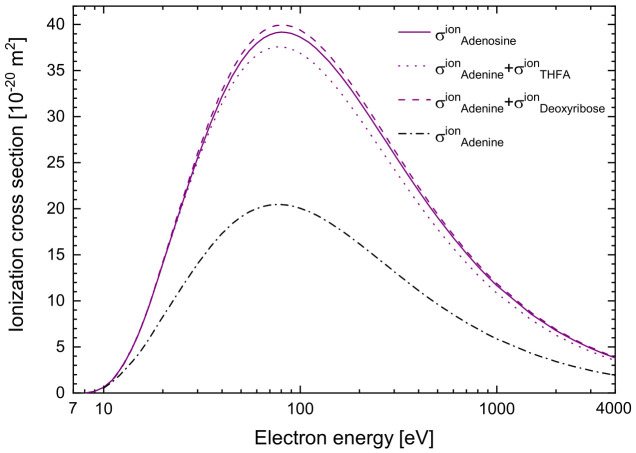
Comparison of the electron impact ionization cross-section for Adenosine and adenine molecules. Estimated ionization cross-sections for Adenosine, obtained as the sum of the ionization cross-sections for adenine and α-tetrahydrofurfuryl alcohol, and for adenine and deoxyribose, are also presented.

**Figure 8:**
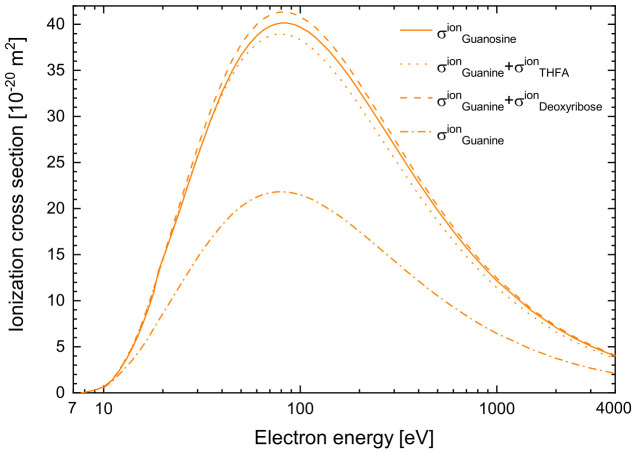
Comparison of the electron impact ionization cross-section for Guanosine and guanine molecules. Estimated ionization cross-sections for Guanosine, obtained as the sum of the ionization cross-sections for guanine and α-tetrahydrofurfuryl alcohol, and for guanine and deoxyribose, are also presented.

The following figures, Figure 5, Figure 6, Figure 7 and Figure 8, present a comparison of the cross-sections of the same types for the corresponding nucleoside (Thymidine, Cytidine, Adenosine, and Guanosine) with the ionization cross-section of the corresponding DNA bases (thymine, cytosine, adenine, and guanine) [37] and the ionization cross-sections obtained as the sum of the ionization cross-sections for the base [37] and α-tetrahydrofurfuryl alcohol [36] and the base [37] and deoxyribose, respectively.

Qualitatively, the ionization cross-sections obtained as the sum of the ionization cross-sections for the DNA/RNA base and tetrahydrofurfuryl alcohol, as well as for the base and deoxyribose, do not differ significantly from the cross-section calculated for the given nucleoside. However, there are quantitative differences. The ionization cross-section, estimated as the sum of the ionization cross-sections of the base and tetrahydrofurfuryl alcohol, is lower than the cross-section for the nucleoside across the entire energy range under consideration. The situation is slightly different for the ionization cross-section, which is estimated as the sum of the ionization cross-sections of the base [37] and deoxyribose. In the vicinity of the ionization threshold up to about 20–30 eV, this cross-section is nearly identical to the ionization cross-section of the corresponding nucleoside. Also, for high ionizing electron energies above 1 keV, both cross-sections have similar values. For energies in the range of 30 eV to 1 keV, this summed cross-section exceeds the one calculated directly. The largest differences occur near the ionization maximum but do not exceed 5%.

Therefore, although the ionization cross-sections for nucleosides, estimated as the sum of the ionization cross-sections for simpler molecular fragments and their corresponding analogs, are in good qualitative agreement with the results of direct calculations, the quantitative values of these cross-sections may be slightly underestimated or overestimated. Therefore, the potential use of such simple summation rules in the modeling of the interactions of the secondary electrons with complex biomolecules requires caution and necessitates an estimation of the potential uncertainty associated with this simple summation rule.

## 3. Theoretical and Computational Methods

The theoretical method and numerical procedures employed in this study are essentially identical to those used in our previous research, in which the cross-sections for collision-induced ionization by electrons were calculated for the DNA and RNA bases and for analogs of other components of deoxyribonucleic acid [36,37], as well as for other simple molecules of significant biological importance, such as formic acid and acetic acid [38], pyridazine [39], pyridine [40], and, more recently, pyrimidine and its halogenated derivatives [41]. For this reason, we will provide a concise description of the method here, along with the necessary details regarding the calculations used in this study.

For the electron impact ionisation cross-section (ICS) calculations, we used the well-known semi-empirical binary-encounter-Bethe (BEB) method [42,43]. The BEB method is a simplified version of the binary-dipole-encounter (BED) method [42] developed by combining the theory of binary collisions [44], applicable in the range of low-energy incident electrons, with Bethe theory, which is valid for high-energy electron collisions [45]. Despite its semi-empirical nature, the BEB method contains no arbitrary parameters and all the quantities required for calculating the ICS are well-defined physical quantities. It is significant that these quantities can be calculated or estimated quite accurately using standard quantum chemistry methods and tools. This method allows us to calculate reliable ionization cross-sections for molecules. Discrepancies between the ionization cross-sections (ICS) calculated using the BEB method and experimental data typically do not exceed ±15% [28,29,30]. The accuracy of the calculated ionization cross-sections largely depends on the quality of the quantum chemical calculations of the physical parameters required by the model. Consequently, the quality of the resulting ICSs depends largely on the level of the theory employed and the basis sets used.

According to the BEB model, the electron impact ionization cross-section for a given single molecular orbital is related to the electron binding energy, *B*, the kinetic orbital energy, *U*, the energy of the impinging electron *T*, and the orbital occupation number, *N*, by the following formula:

(1) $$\sigma^{BEB}=\frac{S}{t+u+1}\left[\frac{\ln t}{2}\left(1-\frac{1}{t^{2}}\right)+1-\frac{1}{t}-\frac{\ln t}{t+1}\right],$$

where u=U/B, t=T/B, $S=4\pi a_{0}^{2}NR^{2}/B^{2}$, $a_{0}=0.5292$ Å, and R=13.61 eV. Given the ionization cross-sections, $\sigma^{BEB}$, for all individual molecular orbitals (MOs), the total electron impact ionization cross-section, $\sigma^{\mathrm{ion}}$, is calculated as the sum of these cross-sections:

(2) $$\sigma^{\mathrm{ion}}=\sum_{i=1}^{n_{MO}}\sigma_{n_{MO}}^{BEB}$$

Quantum chemistry software packages are typically used to calculate the electron binding energy *B*, the kinetic energy of all molecular orbitals *U*, and the orbital occupation numbers *N*. In this study, these quantities were calculated using the Gaussian program [46]. First, the geometries of the molecules under study in their ground state were optimized using the Hartree–Fock (HF) method with the 6-311G(d) and 6-311+G(d) Gaussian basis sets for nucleosides and deoxyribose, respectively. The second step involved single-point HF energy calculations using the same 6-311G(d) and 6-311+G(d) Gaussian basis sets and the obtained geometries of the studied molecules.

Since energies obtained via Koopmans’s theorem for the highest occupied molecular orbitals (HOMO) are usually higher than those measured experimentally (even by about 1–3 eV), outer valence Green function calculations of correlated electron affinities and ionization potentials [47,48,49,50] were also performed using the GAUSSIAN code [46].

## 4. Conclusions

The binary-encounter-Bethe method was used to calculate the ionization cross-sections for electron impact ionization of selected nucleosides and deoxyribose over a wide range of collisional energies. For deoxyribose, satisfactory agreement was observed with calculations performed using the improved binary-encounter-dipole. The obtained ionization cross-sections for the nucleosides studied are in acceptable agreement with the results of calculations performed using the complex optical potential method. It has been demonstrated that the ionization cross-sections of the studied nucleosides can be estimated with considerable accuracy based on the sum of the ionization cross-sections of their respective bases and deoxyribose or α-tetrahydrofurfuryl alcohol.

## Figures and Tables

**Table 1:** Ionization cross-section for electron impact ionization of Deoxyribose, Uridine, Thymidine, Cytidine, Adenosine, and Guanosine in units of $10^{-20}$ m^2^.

Energy [eV]	Deoxyribose	Uridine	Thymidine	Cytidine	Adenosine	Guanosine
7.671						0.00000
8						0.06791
8.057					0.00000	
8.352			0.00000			
8.368				0.00000		
8.627		0.00000				
9		0.05720	0.1088	0.1034	0.1866	0.2877
9.992	0.00000					
10	0.00070	0.2200	0.3116	0.5140	0.6182	0.6563
11	0.1356	0.5990	0.8079	1.217	1.381	1.350
12	0.4439	1.305	1.577	2.123	2.449	2.450
13	0.9050	2.149	2.463	3.150	3.630	3.623
14	1.480	3.094	3.531	4.309	4.945	4.896
15	2.135	4.198	4.783	5.574	6.373	6.311
16	2.865	5.381	6.134	6.949	7.902	7.827
17	3.609	6.630	7.578	8.352	9.458	9.383
18	4.388	7.892	9.011	9.775	11.04	10.94
19	5.181	9.195	10.45	11.20	12.59	13.26
20	5.954	10.47	11.83	12.57	14.07	14.53
22.5	7.724	13.40	14.97	15.70	17.47	17.64
25	9.322	16.03	17.78	18.49	20.52	20.58
27.5	10.73	18.37	20.26	20.96	23.20	23.27
30	11.97	20.42	22.43	23.10	25.54	25.66
35	14.00	23.79	25.97	26.59	29.33	29.63
40	15.53	26.36	28.64	29.24	32.21	32.65
45	16.71	28.35	30.68	31.26	34.39	34.94
50	17.59	29.84	32.19	32.75	35.99	36.63
55	18.24	30.96	33.29	33.85	37.16	37.88
60	18.71	31.77	34.09	34.63	37.98	38.77
65	19.05	32.35	34.63	35.16	38.54	39.39
70	19.28	32.75	34.99	35.52	38.90	39.80
75	19.42	33.00	35.20	35.72	39.10	40.04
80	19.50	33.13	35.29	35.80	39.17	40.14
85	19.52	33.18	35.29	35.79	39.14	40.14
90	19.49	33.14	35.21	35.71	39.03	40.06
95	19.44	33.05	35.08	35.57	38.86	39.91
100	19.35	32.92	34.89	35.38	38.64	39.71
110	19.12	32.54	34.42	34.90	38.08	39.18
120	18.83	32.06	33.86	34.33	37.43	38.55
140	18.16	30.94	32.59	33.04	35.99	37.12
160	17.45	29.74	31.27	31.69	34.50	35.63
180	16.74	28.56	29.97	30.37	33.04	34.15
200	16.06	27.41	28.72	29.11	31.64	32.74
225	15.26	26.06	27.26	27.64	30.02	31.09
250	14.53	24.81	25.93	26.28	28.54	29.58
275	13.85	23.67	24.71	25.05	27.18	28.19
300	13.24	22.62	23.59	23.91	25.94	26.92
350	12.15	20.78	21.63	21.93	23.77	24.69
400	11.23	19.21	19.98	20.25	21.95	22.81
450	10.44	17.87	18.56	18.82	20.39	21.20
500	9.762	16.71	17.35	17.59	19.05	19.82
600	8.648	14.81	15.36	15.57	16.85	17.55
700	7.773	13.32	13.80	13.99	15.14	15.77
800	7.068	12.11	12.54	12.72	13.75	14.33
900	6.487	11.12	11.50	11.67	12.62	13.15
1000	6.000	10.29	10.64	10.79	11.66	12.16
1500	4.396	7.540	7.787	7.897	8.535	8.908
2000	3.496	5.999	6.191	6.278	6.784	7.083
2500	2.916	5.005	5.162	5.235	5.656	5.907
3000	2.509	4.307	4.441	4.503	4.865	5.082
3500	2.207	3.789	3.906	3.960	4.278	4.469
4000	1.973	3.387	3.491	3.540	3.824	3.995

## Data Availability

The original contributions presented in this study are included in the article. Further inquiries can be directed to the corresponding author.

## Abbreviations

The following abbreviations are used in this manuscript:

BEB	Binary-encounter-Bethe
BED	Binary-encounter-dipole
HF	Hartree–Fock
ICS	Ionization cross-section
OVGF	Outer valence Green function
THF	tetrahydrofuran
THFA	α-tetrahydrofurfuryl alcohol
